# Impaired Attentional Processing During Parabolic Flight

**DOI:** 10.3389/fphys.2021.675426

**Published:** 2021-05-13

**Authors:** Anika Friedl-Werner, Marie-Laure Machado, Costantino Balestra, Yannick Liegard, Bruno Philoxene, Katharina Brauns, Alexander C. Stahn, Martin Hitier, Stephane Besnard

**Affiliations:** ^1^Charité – Universitätsmedizin Berlin, a Corporate Member of Freie Universität Berlin and Humboldt-Universität zu Berlin, Institute of Physiology, Center for Space Medicine and Extreme Environments Berlin, Berlin, Germany; ^2^Université de Normandie, INSERM U1075 COMETE, Caen, France; ^3^Environmental, Occupational & Ageing “Integrative Physiology” Laboratory, Haute Ecole Bruxelles-Brabant, Brussels, Belgium; ^4^DAN Europe Research Division (Roseto (It)-Brussels (B)), Brussels, Belgium; ^5^Unit of Experimental Psychiatry, Department of Psychiatry, Perelman School of Medicine at the University of Pennsylvania, Philadelphia, PA, United States; ^6^Department of Otolaryngology Head and Neck Surgery, Centre Hospitalier Universitaire de Caen Normandie, Caen, France; ^7^Department of Anatomy, Université de Normandie, Caen, France; ^8^Aix Marseille Université, CNRS, UMR 7260, Laboratoire de Neurosciences Sensorielles et Cognitives - Equipe Physiopathologie et Thérapie des Désordres Vestibulaires, Marseille, France

**Keywords:** microgravity, attention, scopolamine, anxiety, human, adverse effects

## Abstract

Previous studies suggest that altered gravity levels during parabolic flight maneuvers affect spatial updating. Little is known about the impact of the experimental setting and psychological stressors associated with parabolic flight experiments on attentional processes. To address this gap, we investigated the level of alertness, selective and sustained attention in 1 and 0 g using a Go/No-Go Continuous Performance Task. We also identified several parameters associated with the experimental set-up of a parabolic flight that could be expected to affect attentional processing. These included the use of scopolamine, sleep quality prior to the flight day, participant’s stress level as well as mood and anxiety state before and after the parabolic flight. We observed a deterioration in attentional processing prior to the first parabola that was further aggravated in weightlessness and returned to baseline after the last parabola. *Reaction Time*, *Hit* and *False Alarm Rate* were moderately correlated with self-reported anxiety state, but not cortisol levels or emotional states. The use of scopolamine had minor effects on *Reaction Time*. Our results confirm previous studies reporting impairments of cognitive performance in 0 g, and highlight important aspects that should be considered for the design of behavioral research experiments in future parabolic flight campaigns.

## Introduction

With the resurgence of interest in space exploration and human settlement in space, researchers are seeking to better understand the effects of gravity on the human body and to ensure safe and successful space exploration. The central nervous system has been brought into focus of these investigations, and there is a growing interest in better understanding the effects of spaceflight on brain and behavior (Roberts et al., 2020). Recent studies have reported structural brain changes using magnetic resonance imaging (MRI) after prolonged space flight (Demertzi et al., 2016; Roberts et al., 2017; Van Ombergen et al., 2018, 2019) and alterations in functional connectivity after exposure to different gravity conditions, i.e., short periods of hyper- and hypogravity during parabolic flights (Van Ombergen et al., 2017). Evidence from spaceflight research has also reported that weightlessness led to altered spatial cognition abilities (Paloski et al., 2008; Cheron et al., 2014), and impaired sensory-motor integration and control (Casellato et al., 2012; Hallgren et al., 2016; Reschke et al., 2017). Likewise, Stahn et al. (2020) and others have shown that spatial cognition is significantly impaired during altered gravity conditions (Grabherr et al., 2007; Grabherr and Mast, 2010; Clément et al., 2016). In contrast to these studies, Wollseiffen and colleagues reported faster reaction times for a complex mental arithmetic task (Wollseiffen et al., 2016) as well as in combination with an oddball task paradigm (Wollseiffen et al., 2019) in microgravity during parabolic flight. To fully understand the effects of altered gravity conditions on neurobehavioral performance, it is important to disentangle the effects related to microgravity from potential confounders associated with parabolic flight maneuvers *per se*. Factors such as an increased stress and anxietly level, especially for first-time flyers, and poor sleep prior to the flight day may impact behavioral measures. Further, participants may also experience severe motion sickness that is typically attenuated by an antiemetic drug administered before the flight. A lack of sleep and poor sleep quality, antiemetic drugs, mood, and stress, each of which can confound cognitive and motor performance (Wesnes and Warburton, 1983; Lim and Dinges, 2008; Bestaven et al., 2016), functions that are known to be dependent on the level of attention and selective attention abilities (Carrasco, 2018; Ruff and Cohen, 2019; Song, 2019).

Here, we investigated the level of alertness, selective and sustained attention using a Go/No-Go Continuous Performance Task (CPT) in male first-time flyers before, during, and after parabolic flight exposure. As a secondary outcome, we assessed parameters associated with the experimental set-up of the parabolic flight campaign that we expected to affect attentional processing, i.e., the use of an antiemetic drug, stress level, participant’s mood and anxiety state as well as their sleep quality prior to the flight. All paradigms and questionnaires have been validated in previous behavioral research (Rosvold et al., 1956; McNair et al., 1981; Spielberger et al., 1983; Drummond et al., 2005; Golding et al., 2017) and were adapted when necessary to the parabolic flight constraints. We hypothesized that the level of alertness and attention is impaired by the weightlessness as well as by the experimental set-up of the parabolic flight campaign itself, and that both factors would influence cognitive performance.

## Materials and Methods

### Participants

Twelve men (mean age: 48.75 years ± 8.7, range: 34–55 years) participated in the study. All participants were naïve to the experience of microgravity, non-smokers, free of any cardiovascular, vestibular, psychiatric, and neurological disorders, had a normal or corrected-to-normal vision, and passed a Class 3 Aviation medical exam. Approximately 75–90 min prior to the take-off, all participants received 0.175 mg of scopolamine that was injected subcutaneously by the campaign’s flight physician.

### Study Design

**FIGURE 1:**
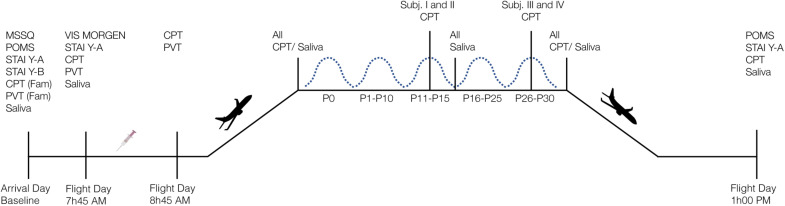
Schematic overview of the study design. Four participants were tested on each flight day. MSSQ, Motion Sickness Susceptibility Questionnaire; POMS, Profile of Mood States; STAI Y-A/Y-B, State-Trait Anxiety Inventory Form Y Part A/B; CPT, Continuous Performance Task; PVT, Psychomotor Vigilance Task, Fam, familiarization session; VIS Morgen, sleep questionnaire; P, Parabola; syringe represents subcutaneous scopolamine injection.

Data were collected on board of an Airbus 310 Zero-G during the 131^st^ parabolic flight campaign operated by Novespace^1^. The campaign took place in October 2017 in Bordeaux Merignac, France, and was sponsored by the Centre national d’étude spatiales (CNES). The campaign was composed of a familiarization day that was used to collect baseline data, followed by three days of parabolic flights. Each flight started in the morning at about 9h30 am, and was finished at about 1 pm. The flight consisted of 31 parabolas, each starting and ending with a hypergravity phase of 1.8 g of approximately 20 s, and a microgravity^2^ phase of approximately 22 s in-between. Averaged values of the hypogravity periods were 0.0095 g, 0.0036 g, and 0.004 g for x-, y-, and z-axis, respectively. The averaged g-levels during each parabola are provided in Supplementary Table 1. All participants were familiarized with the test protocol on the first day of the campaign and participated in one parabolic flight, i.e., on one of the three consecutive flight days. On the flight day, participants were offered an antiemetic drug (scopolamine) as a voluntary routine option because scopolamine has been shown to decrease the risk of motion sickness compared with no medication intake during parabolic flights (Golding et al., 2017). All participants volunteered to received scopolamine in the morning prior to the flight. The experiment was approved by the Comité de Protection des Personnes Nord Ouest III, Caen, France (HYPOCAMPUS 2015-A02014-45) and conformed to all standards of human research set out in the declaration of Helsinki. All participants were informed about the purpose, experimental procedures, and the risks before giving their verbal and written informed consent. Four participants were tested per flight day. Figure 1 displays a schematic overview of the study design.

### Inflight-Testing

**FIGURE 2:**
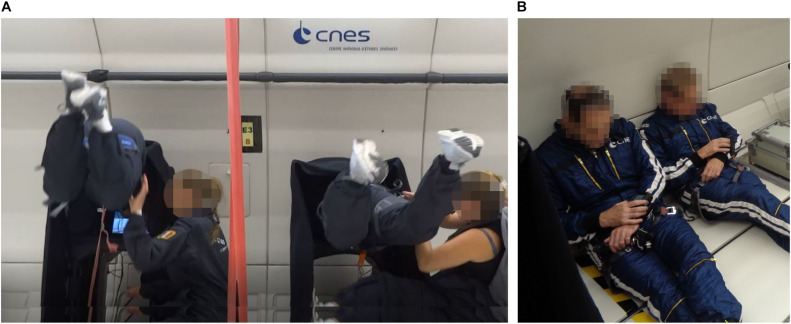
Experimental set-up. **(A)** Participants performing the Continuous Performance Task (CPT) in a controlled free-floating position. **(B)** Participants performing the psychomotor vigilance test (PVT) using a diving computer that was attached to participants’ wrist. During both tasks, two participants were tested at the same time.

*Go/No-Go Paradigm.* We administered a Go/No-Go Continuous Performance Task (CPT) for recording selective and sustained attention as well as impulsive behavior. The test has been introduced by Rosvold et al. (1956) and, since then, has been implemented in different forms in the research on attentional processes. Single letters were presented in random order on the computer screen. Every 920 ms a letter appeared and was displayed for the same duration. During the 0 g condition, participants were asked to react to a specific target letter (X) by pressing the space bar with their index as soon as the target appeared and withhold responses to all other stimuli. Targets appeared in 30% of the trials, i.e., 7 targets of 22 trials per parabola. The task was programmed and applied using the VRmaze software (Machado et al., 2019), adapted and shortened according to the requirements and time constraints of the parabolic flight maneuver, and presented on a 15-inch laptop (ZBook 15 G5 Mobile Workstation, Hewlett Packard). Cognitive data were collected at the following points in time: (1) once during familiarization session on the day of arrival at Novespace; (2) on the flight day before and after scopolamine injection; (3) inflight at 1 g before experiencing the first parabola, during 0 g, and inflight at 1 g after the last parabola; and (4) post-flight (Figure 1). Data collected at the time points before and after scopolamine injection were used to evaluate the effect of antiemetics (see section “Scopolamine” for further information). For the analysis of the data collected inflight during 1 and 0 g, parameters obtained post-medication were used as a baseline to avoid any further bias. Data were acquired in a seated position during 1 g onboard of the plane and in a controlled free-floating position during 0 g condition (Figure 2A). A fabric-covered rack was used to minimize visual distractions throughout all conditions. Participants performed the CPT either from parabola 11 to 15 or from parabola 26 to 30 (Figure 2). During the remaining parabolas the participants performed a virtual navigation task, which will be reported elsewhere. Performance was quantified by (1) *Reaction Time* (RT) for target trials in milliseconds (ms); (2) *Hit Rate* (correct detection of the target letter) in percentage to determine attentional capacity; and (3) *False Alarm Rate* (Reaction to non-targets) in percentage as an indicator of impulsivity. Additionally, we also computed *d-prime* (d’) as an indicator of sensitivity. The *Omission Error Rate* in percent as an indicator of distraction is reported descriptively, because the inferential statistical characteristics are identical to those of the *Hit Rate*.

### Testing of Factors Associated With the Experimental Set-Up of the Parabolic Flight Campaign

#### Motion Sickness

To evaluate participant’s susceptibility to motion sickness, we administered the short form of the Motion Sickness Susceptibility Questionnaire (MSSQ) on the day of arrival. The questionnaire has been validated and previously used during parabolic flight campaigns (Golding et al., 2017). By assessing previous experience of motion sickness symptoms and nausea in different transport modes during child- and adulthood, a raw score between 0 and 54 is calculated and a percentile conversion is given (Golding, 2006). Irrespective of the test result, an antiemetic drug was offered to all participants of the experiment to avoid severe motion sickness symptoms.

#### Scopolamine

Scopolamine is a muscarinic antagonist that is often used for preventing nausea and motion sickness symptoms (Lochner and Thompson, 2016) but also provokes drowsiness and fatigue and has been reported to disrupt performance in tests of sustained attention (Wesnes and Warburton, 1983). During parabolic flight campaigns, scopolamine is offered as a routine to avoid severe motion sickness symptoms that may occur in up to 90% of first-time flyers. The sedative and antiemetic effect of scopolamine occurs approximately 30 min after medication. To identify whether scopolamine impacts participants’ attentional processing, we also administered the CPT on the flight day before and 30 min after subcutaneous scopolamine injection. Additionally, we also employed a Psychomotor Vigilance Task (PVT) before and after medication to assess participants’ vigilance. The test has been validated by means of functional magnetic resonance imaging (fMRI) (Drummond et al., 2005). A visual stimulus in form of a red dot appeared ten times at random interstimulus intervals throughout a total test duration of 2 min. Participants were asked to press a button as quickly as possible each time the red dot would appear and *Reaction Time* was recorded. The PVT was an adapted version of the test described by Moore et al. (2017) and was administered using a modified diving computer (MARES Icon^®^) that was attached to participants’ wrist (Balestra et al., 2018). The test was performed in a seated position in an open space area in the aircraft (Figure 2B).

#### Stress

Salivary cortisol was collected using the Salivette^®^ (Sarstedt, Nümbrecht, Germany) cotton swab system to assess participant’s stress response. All participants were familiarized with the correct sample collection, i.e., avoid eating, drinking, and brushing teeth at least 30 min prior to sample collection, and chew on the cotton swab for 60 s. Saliva samples were collected on the day of arrival before noon, in the morning of the flight day after wake-up, before experiencing the first parabola (P0), after the 15^th^ and 30^th^ parabola (P15, P30), and post-flight. Samples were subsequently frozen and stored at a temperature of −25°C. Cortisol concentrations were then quantified by an electrochemiluminescence immunoassay (ECLIA, Roche, Mannheim, Germany) on a *cobas e411* analyzer at the University Hospital of Caen, Normandy, France. To verify whether cortisol levels were not affected by sleep quality, we also calculated the change in salivary cortisol from P0 to P30 and correlated this change with self-reported sleep quality and total sleep duration (see also section “Sleep Quality” for sleep assessment).

#### Anxiety and Mood State

Anxiety level was assessed using the French version (Form Y) of the State-Trait Anxiety Inventory (STAI) (Spielberger et al., 1983). Trait anxiety as a personal characteristic was determined only once on the day of arrival. State anxiety was assessed three times: on the day of arrival at Novespace, before take-off, and after landing. To evaluate changes in mood states, a validated French version of the Profile of Mood States Questionnaire (POMS) (Cayrou et al., 2003) was administered on the day of arrival and after the parabolic flight.

#### Sleep Quality

To evaluate participants’ sleep quality and sleep duration of the preceding night of the parabolic flight, the VIS-Morgen Questionnaire was administered. The questionnaire was originally introduced by the *Centre du Sommeil et de la Vigilance, Hôpitaux universitaires*, *Paris Centre* and assesses sleep quality and participant’s energy upon awakening on a visual analog scale between 0 and 10 (Dubois et al., 2013). It also records the number of perceived wake-ups and sleep duration. The questionnaire was administered on the flight day before boarding the plane.

### Association Between CPT Performance and Factors Associated With the Experimental Set-Up of a Parabolic Flight Campaign

To identify the relationships between attentional processes and emotional state, anxiety, and stress, we performed an exploratory analysis and correlated CPT data (*Reaction Time, Hit Rate, False Alarm Rate*) collected during 1 g inflight (before P0 and after P30) with salivary cortisol (before P0 and after P30), and with mood states (POMS questionnaire administered on-ground immediately before and after flight). We also correlated CPT data (on the day of arrival, before P0, and after P30) with state anxiety score (on the day of arrival, on-ground immediately before and after parabolic flight exposure) at all three time points.

### Statistical Analysis

Descriptive statistics are presented as marginal means and standard errors of the mean (*SE*) unless stated otherwise. Differences between points in time were assessed using a linear mixed model with *Time* as a fixed factor, and *Subject* as a random factor (random intercept only). Pre-planned contrasts were computed for simple comparisons between points in time with a sequential Holm – Bonferroni correction for multiple comparisons (Holm, 1979). Effect sizes are reported as Cohen’s *d* and 95% confidence intervals (CI). The relationships between CPT variables and mood states, anxiety, and stress were determined using repeated measures correlation. The level of significance was set at α = 0.05 (two-sided) for all tests. Estimated marginal means were calculated using emmeans package (Lenth, 2016), effect size and confidence intervals were computed using psych package, the sensitivity index d’ was calculated using psycho package, version 0.5.0, correlation analysis was performed using rmcorr package (Bakdash and Marusich, 2017), and figures were created using ggplot2 (Wickham, 2016). All statistical analyses and graphical illustrations were carried out using the software package R (R Core Team, 2018).

## Results

### Inflight Testing

**FIGURE 3:**
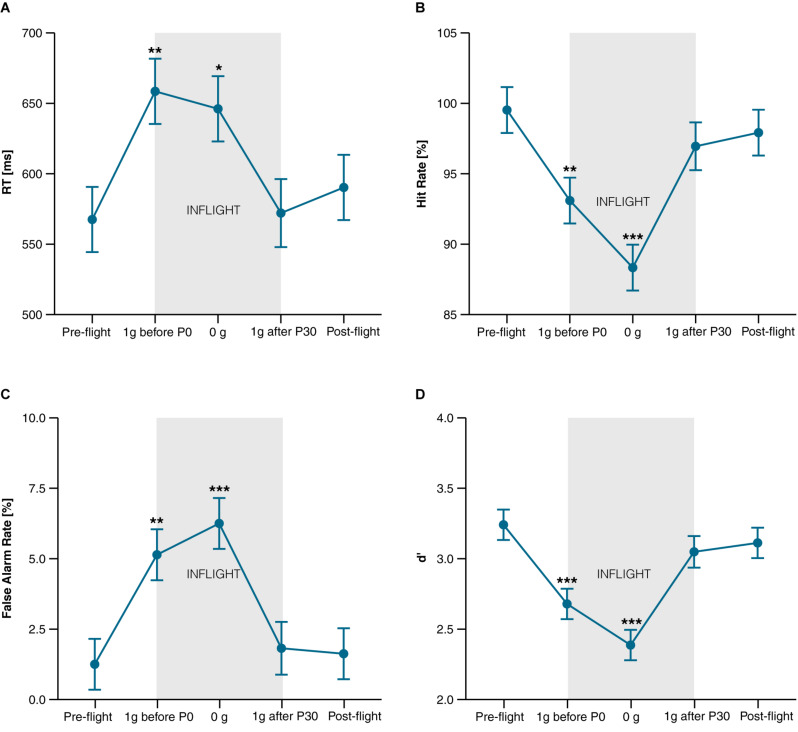
CPT performance. **(A)** Reaction Time of target stimuli in ms; **(B)** Hit Rate (correct reactions to target stimuli) in percentage; **(C)** False Alarm Rate (reactions to non-targets) in percentage; **(D)** d’ (indicator for task sensitivity). Data is presented as marginal means ± SE and was collected at the following points in time: Pre-flight (30 min after scopolamine injection), inflight at 1 g before the first parabola (1 g before P0.), during microgravity (0 g), at 1 g after the last parabola (1 g after P30), and after landing (Post-flight). *P < 0.05, **P < 0.01, ***P < 0.001 compared to pre-flight.

Compared to pre-flight, a deterioration in task performance was observed in 1 g before experiencing the first parabola (1 g before P0) and during 0 g, but not in 1 g after the last parabola (1 g after P30) and post-flight. The decline in task performance was characterized by a significantly longer *Reaction Time* [*t*_43.2_ = 2.98, *P* = 0.019, *d* = 0.86 (0.18, 1.52) and *t*_43.2_ = 2.58, *P* = 0.040, *d* = 0.74 (0.09, 1.37)], by a lower *Hit Rate* [*t*_43.1_ = −2.93, *P* = 0.016, *d* = −0.85 (−1.50, −0.17) and *t*_43.1_ = −5.11, *P* < 0.001, *d* = −1.47 (−2.29, − 0.63)], and by a higher rate of *False Alarms* [*t*_42.9_ = 3.66, *P* = 0.002, *d* = 1.06 (0.33, 1.76) and *t*_42.9_ = 4.71, *P* < 0.001, *d* = 1.36 (0.55, 2.14)] during 1 g before P0 and during 0 g, respectively (Figures 3A–C). At all points in time, d’ exceeded a value of two, suggesting that participants were generally able to discriminate the signal over noise. In line with the performance decline, d’ also decreased significantly in 1 g before P0 [*t*_42.9_ = −4.31, *P* < 0.001, *d* = −1.24 (−1.99, −0.47)] and during 0 g [*t*_42.9_ = −6.54, *P* < 0.001, *d* = −1.89 (−2.84, −0.91)], and returned to baseline (pre-flight) in 1 g after P30 and post-flight (both *P*s > 0.3) (Figure 3D). Numerically, *Hit* and *False Alarm Rates* were also further impaired in 0 g compared to 1 g before P0 resulting in a significantly lower d’ [*t*_42.9_ = −2.23, *P* < 0.031, *d* = −0.64 (−1.26, −0.01)]. Comparisons of all points in time of the different task variables are provided in Supplementary Table 2.

### Factors Associated With the Experimental Set-Up of a Parabolic Flight Campaign

#### Motion Sickness

The MSSQ score of 5 ± 4.4 revealed that participants were less susceptible to intrinsic motion sickness than the general population (MSSQ percentile: 23 ± 18.4 vs. a norm of 50). One participant experienced discomfort during the flight after inflight data collection was completed. Due to continued discomfort after landing, post-flight data could not be collected for this participant.

#### Scopolamine

All participants received scopolamine in the morning prior to the flight. Table 1 shows the parameters of CPT and PVT before and 30 min after subcutaneous injection of scopolamine. After scopolamine injection, RT was longer for the CPT and PVT, but only significant for the latter [*F*_1,11.8_ = 8,31, *P* = 0.014, *d* = 0.83 (0.16, 1.48)]. Numerically, a lower *False Alarm Rate* was observed after the medication for the CPT, but not the PVT. Longer RTs in the CPT were accompanied by a higher *Hit Rate* (both *P*s > 0.3) suggesting a strategy change. To control for a speed-accuracy tradeoff, we reanalyzed *Hit Rate* of CPT using RT as a covariate. After correction the improvement in *Hit Rate* from pre to post-medication was still discernable, though not significant (*P* = 0.089).

**TABLE 1:** CPT and PVT performance before and after scopolamine injection*.

	Pre-medication	Post-medication	*DF*_1_, *DF*_2_	*F*	*P*
**CPT**					
RT (ms)	542 (21.1)	568 (21.1)	1, 11	0.91	0.360
Hit Rate (%)	99.0 (0.4)	99.5 (0.4)	1, 11	1.00	0.339
False Alarm Rate (%)	1.78 (0.5)	1.22 (0.5)	1, 22	0.58	0.455
**PVT**					
RT (ms)	307 (10.5)	339 (10.5)	1, 11.8	8.31	0.014
Hit Rate (%)	97.5 (1.46)	95.9 (1.46)	1, 11.6	1.81	0.204
False Alarm Rate (%)	na	na	na	na	na

*Data represent marginal means and standard error in parentheses. CPT, Continuous Performance Task; PVT, Psychomotor Vigilance Task; RT, Reaction Time in ms; Hit Rate, correct reaction to target stimuli in percentage; False Alarm Rate, reaction to non-targets in percentage; *DF*_1_, numerator degrees of freedom; *DF*_2_, denominator degrees of freedom; *F*, F-statistics; *P*, p-value. Data were obtained before and after 30 min of a subcutaneous scopolamine injection of 0.175 mg.

#### Stress

**FIGURE 4:**
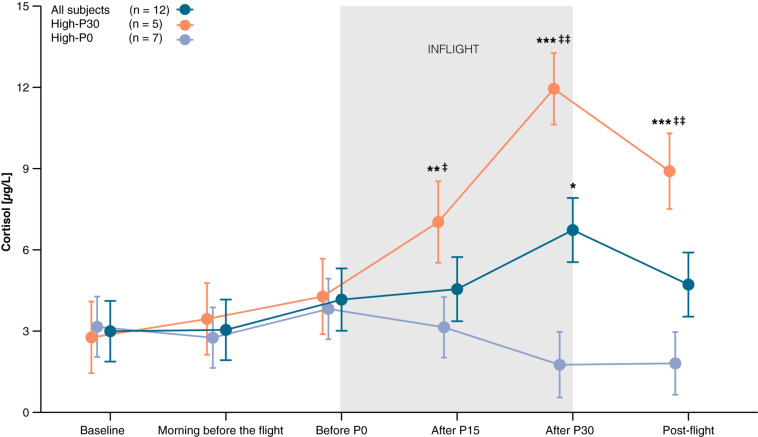
Time course of salivary cortisol levels. Baseline saliva was collected between 9 am and noon on the day of arrival. Two different patterns could be observed, with the highest measured cortisol either after P30 (High-P30) or before P0 (High-P0). Orange line represents participants of High-P30, violet line represents participants of High-P0, dark blue line shows data of all participants. Data are presented as marginal means ± SE. *P < 0.05, **P < 0.01, ***P < 0.001 compared to baseline, ^‡^*P* < 0.05, ^‡‡^*P* < 0.001 compared to High-P0.

On average, cortisol levels increased from baseline throughout the parabolic flight reaching a peak after the 30^th^ parabola before decreasing again at post-flight (effect of *Time*: *F*_5,48.3_ = 2.61, *P* = 0.036). Pre-planned contrasts revealed that cortisol concentrations measured after the last parabola (after P30) were significantly higher compared to baseline [*t*_48.3_ = 3.14, *P* = 0.014, *d* = 0.91 (0.21, 1.57)]. Visual inspection of the data revealed two types of responders, those whose cortisol peaked after the last parabola (High-P30), and those who reached their highest cortisol level before the first parabola (High-P0). Figure 4 shows the time course of salivary cortisol concentrations for all subjects and for each subgroup. Compared to baseline, salivary cortisol levels of High-P30 increased significantly throughout the parabolic flight [P15: *t*_43.6_ = 3.16, *P* = 0.009, *d* = 25.50 (8.83, 41.62) and P30: *t*_43_ = 8.05, *P* < 0.001, *d* = 25.18 (8.71, 41.09)], and decreased post-flight. For High-P0, only slight changes in cortisol levels that were not significant were observed. Accordingly, group differences between High-P30 and High P-0 were observed at P15, P30, and post-flight [*t*_25.2_ = −2,07, *P* = 0.049, *d* = 14.75 (5.05, 24.11), *t*_22_ = −5.68, *P* < 0.001, *d* = 12.88 (4.39, 21.56), *t*_22.7_ = −3.91, *P* < 0.001, *d* = 13.31 (4.54, 22.03) respectively]. The changes in cortisol concentrations obtained prior to the first and after the last parabola were associated with self-reported sleep quality (Spearman’s ρ = 0.79, *P* = 0.012), but not with total sleep duration (ρ = 0.56, *P* = 0.117).

#### Anxiety and Mood States

Participants were characterized by a low trait anxiety score of 34.2 ± 3.5 (range: 26–39) relative to the norm. State anxiety changed throughout the parabolic flight campaign, increasing from 25.9 ± 1.9 on the day of arrival (baseline) to 28.5 ± 1.9 on the morning before the flight, and decreasing below baseline to 23.8 ± 2.03 post-flight (effect of *Time*: *F*_2, 20.38_ = 2.63, *P* = 0.097). Pre-planned contrasts showed that the decrease in anxiety from pre-flight to post-flight was close to statistical significance [*t*_20.6_ = 2.27, *P* = 0.068, *d* = 0.66 (0.02, 1.27)]. Furthermore, lower scores in the subscales of tension-anxiety and anger-hostility of the POMS questionnaire were observed after parabolic flight exposure [*F*_1,11_ = 5.62, *P* = 0.037, *d* = −0.68 (−1.3, −0.04) and *F*_1,11_ = 9.67, *P* = 0.010, *d* = −0.9 (−1.56, −0.21) respectively]. Total mood disturbance (TMD) decreased from 0.96 ± 4.49 to −6.13 ± 4.49, nearly reaching statistical significance [*F*_1,11_ = 4.46, *P* = 0.058, *d* = −0.61 (−1.22, 0.02)]. There were no significant changes in the scores of depression, vigor, fatigue, and confusion (all *P*s > 0.23). Table 2 shows a detailed overview of participants’ mood states before and after the flight.

**TABLE 2:** Participants’ Profile of Mood States (POMS) before and after the parabolic flight*.

	Pre-flight	Post-flight	*F*_1,11_	*P*	Effect size (95% CI)
Tension	5.83 (1.14)	3.46 (1.14)	5.62	0.037	–0.68 (–1.3, –0.04)
Depression	1.92 (0.56)	1.67 (0.56)	0.20	0.667	–0.13 (–0.69, 0.44)
Anger	5.42 (1.04)	2.04 (1.04)	9.67	0.010	–0.9 (–1.56, –0.21)
Vigor	20.90 (1.02)	22.20 (1.02)	1.56	0.238	0.36 (–0.23, 0.94)
Fatigue	4.37 (1.35)	5.44 (1.35)	0.66	0.432	0.24 (–0.34, 0.8)
Confusion	4.33 (1.27)	3.42 (1.27)	1.61	0.231	–0.37 (–0.94, 0.23)
TMD	0.96 (4.49)	−6.13(4.49)	4.46	0.058	–0.61 (–1.22, 0.02)

*Data represent marginal means and standard error in parentheses. TMD, Total Mood Disturbance, *F*, F-statistics; *P*, p-value; Effect size is Cohen’s d in 95% confidence intervals (95% CI).

#### Sleep Quality

The participants slept approximately 6h20min ± 1 h (range between 5 and 8 h) the night before the parabolic flight with self-reported sleep quality of 7.4 ± 1.7 (visual analog scale between 0 and 10 with 10 indicating the highest sleep quality). Furthermore, participants reported that they woke up approximately once during the night, and having a generally good state of mind (visual analog scale: 8.3 ± 1.3).

### Correlation Between Cognitive Performance, Cortisol, and Emotional States

A similar time course of participant’s salivary cortisol levels and state anxiety was observed compared to the time course of CPT performance parameters. Therefore, we investigated the relationships between state anxiety, cortisol, and mood states (Anger, Tension, and TMD) with participant’s CPT performance using repeated measures correlation. A higher level of anxiety was associated moderately with slower *Reaction Time* (*r* = 0.52, *P* = 0.019), lower *Hit Rate* (*r* = −0.4, *P* = 0.06), and higher *False Alarm Rate* (*r* = 0.60, *P* = 0.005). We did not find any significant correlation between cortisol concentrations and CPT parameters (all *P*s > 0.182) and between mood states and CPT (all *P*s > 0.407).

## Discussion

For further space missions, it is important to identify the neurobehavioral implications of weightlessness and transitions between gravity levels. Parabolic flight maneuvers provide a unique opportunity to assess the acute effects of hyper- and hypogravity on cognitive performance. Stahn et al. (2020) and others have shown that spatial cognition is significantly impaired during altered gravity conditions (Grabherr et al., 2007; Grabherr and Mast, 2010; Clément et al., 2016). It is unclear to what extend these effects are also observed for other cognitive domains as previous studies have also reported improvements in tasks targeting executive functions (Wollseiffen et al., 2016, 2019).

Here, we investigated the effects of microgravity during parabolic flights on a Go/No-Go Continuous Performance Task. We also aimed to identify potential confounders associated with the experimental setting of parabolic flight experiments. We observed a deterioration in performance of the CPT for both conditions, i.e., before experiencing the first parabola and during the microgravity phase compared to pre-flight testing on-ground characterized by a lower *Hit Rate* and increased *False Alarm Rate* and *Reaction Time*. The performance impairments observed during the flight are likely to be related to various factors associated with the anticipation of the first parabola experience. For instance, compared to the day of arrival (baseline), an increased level of anxiety was reported by the participants immediately before take-off that decreased below baseline post-flight. The changes in anxiety were moderately associated with the changes in *Reaction Time*, *Hit* and *False Alarm Rate* of the CPT.

Additional aspects that may have impinged the attentional capacity on-board are the unfamiliar workload associated with the preparation of the experiment, and alternating the attentional focus between the experiment, pilot announcement, and directions given by the operators and safety crew. The impairments in CPT performance variables observed during 1 g prior to the first parabola were further deteriorated during 0 g, reflecting a gravity effect on attentional processing. We suggest that changes in sensory perception of the own body and in the control of movements in microgravity combined with the emotional state increase the demand of divided attention. All participants were novices to the microgravity experience. Experiencing weightlessness and responding to this novel posture can have considerable impact on the attentional load. The parietotemporal sensory cortex, precuneus, hippocampus as well as subcortical structures such as the thalamus integrate information from the somesthetic, visual, and the vestibular system that support spatial abilities (Besnard et al., 2015; Smith, 2017) including self-perception and one’s position during locomotion, the perception of verticality, mental rotation, orientation, navigation, and spatial memory (Lopez, 2016). The vestibular system is the sensor of terrestrial gravity by its otolithic component and plays a key role in the cortical calibration of visual and somesthetic information related to spatial orientation (Cullen, 2012). The otolithic responses of the vestibular organ are inhibited in weightlessness (Probst et al., 1996; Reschke et al., 2018) and spatial abilities are impaired during hyper- and hypogravity (Mittelstaedt and Glasauer, 1993a, b; Grabherr and Mast, 2010; Stahn et al., 2020). This notion is also supported by a recent functional imaging study, reporting decreases of intrinsic connectivity within the right temporoparietal junction in first-time flyers after parabolic flight (Van Ombergen et al., 2017). Together, these data suggest that vestibular sensory awareness in microgravity phases may play a role in increasing attentional loading on spatial cognitive functions during microgravity phases in which participants are “spatially lost.” Thus, prioritizing self-perception and balance control during microgravity may challenge spatial cognition. The direct effect of the vestibular system on attention remains poorly investigated. It has been shown that vestibular deficiency impaired attention abilities in rodents (Zheng et al., 2009) and humans (Bigelow and Agrawal, 2015) including attention related to visual reward-seeking (Blini et al., 2018). It can be speculated that the decrease in sustained attention for a specific task in microgravity phases is somewhat related to disturbances of the vestibular input and its associated changes in spatial cognition requiring divided attention.

Likewise, the effects of the somesthetic system on attention are currently not well understood. The participants remained secured in a controlled free-floating position that is expected to decrease the somesthetic effect on attention compared to unrestricted free-floating. Additionally, it can also be presumed that the emotional states associated with the sensory perception and the stress-related hormonal effects modulate attention and need to be considered as confounders of attentional control during parabolic flight. The interactions between emotion and attention are well documented (Schultebraucks et al., 2016; Dolcos et al., 2019). Several studies support the role of vestibular inputs for emotional processing (Lopez, 2016; Rajagopalan et al., 2017; Barona-de-Guzmán et al., 2018), including the fear of falling (Schlick et al., 2016) or panic disorders (Perna et al., 2001). It would be worthwhile to further evaluate the attentional abilities of participants with considerable previous parabolic flight experiences. This may allow to discriminate the effects between an acute and an adaptive effect of this particular environment. We expect that a significant history of parabolic flight experience will attenuate the performance decline observed prior to the first parabola. This hypothesis is based on the assumption that frequent flyers are less prone to motion sickness (Golding et al., 2017) and other factors associated with the parabolic flight environment. It is very likely that data of participants with previous flight experience would provide more robust measures of neurobehavioral performance because some of the potential confounding effects are minimized due to the familiarity with the experimental setting and reduced novelty of the g-transitions. In contrast to this hypothesis, Wollseiffen et al. (2016) did not find any differences between experienced and first-time flyers in a complex arithmetic task. They also reported faster reaction times for the highest level of difficulty in the arithmetic task in 0 g compared to 1 g. They attributed the improved performance to the microgravity-induced increases in cerebral blood flow and oxygenation (Blaber et al., 2013; Wollseiffen et al., 2019). Notably, the participants’ responses were also less accurate in 0 g, suggesting a change in response strategy. Thus, it cannot be concluded *per se* that increased cerebral blood flow increases cognitive performance. It can be rather assumed that faster reaction times during altered gravity levels in parabolic flight may also be associated with the experimental conditions of parabolic flight studies, including, but not limited to, performing tasks during bouts of 20 s of weightlessness under considerable time constraints. Additionally, response speed may also vary throughout the flight, independent of the gravity level as observed in the present study.

We also investigated the emotional state using surveys and determined salivary cortisol as an indicator of stress level. Cortisol plays an important role in various physiological processes and is an acceptable marker for stress (Hannibal and Bishop, 2014). The highest cortisol concentration was observed at the end of the parabolic flight. This effect was also reported in previous parabolic flights using serum samples (Schneider et al., 2009). Inspection of individual responses revealed two different phenotypes: one cluster of participants showed their highest cortisol concentrations before the flight that decreased after the last parabola (around noon), and a second cluster those showed the highest cortisol levels after the last parabola. Peak cortisol production usually occurs in the early morning and declines throughout the day with lowest cortisol levels in the late evening and first half of the night (Tsigos and Chrousos, 2002). We conclude that participants showing a decreasing pattern in cortisol levels throughout the flight are less stressed because of the typical circadian pattern of cortisol secretion. In contrast, participants whose cortisol secretion peaked around noon seemed to be more stressed. Additionally, we also observed a strong correlation between self-reported sleep quality and higher cortisol secretion during the flight. However, it is unclear whether the circadian pattern of cortisol secretion was disrupted due to reduced sleep quality, or whether perceived stress was the cause of poor sleep. In the present study, we did not find an association between cortisol concentrations and sleep duration, self-reported anxiety or attention. It is possible that self-reported data on anxiety may be confounded by a response bias such as social desirability and acquiescent in the present cohort, so that the recorded anxiety levels may not have reliably reflected the participants’ true affective states (Kreitchmann et al., 2019). The present data on self-reported anxiety should therefore be interpreted cautiously. We also acknowledge that four out of five participants who showed their cortisol maximum at noon flew on the first day of the campaign. Thus, they may have been more stressed relative to the participants flying on the second or third day of the parabolic flight campaign.

According to the questionnaires, all participants showed an increase in anxiety in the morning of the flight that decreased after the flight. Additionally, we also noted that participants had significantly higher ratings for the subscales “Tension” and “Anger” before the parabolic flight, suggesting an increased arousal and nervousness associated with the uncertainties of the parabolic flight experience. Increases in cortisol levels and self-reported arousal indicate an activation of the sympathetic system (Thau and Sharma, 2019) that may not have been only induced by the novel parabolic flight experience itself, but also by the cognitive task the participants had to complete. Indeed, mental challenges such as mental arithmetic have shown to increase heart rate during different g-levels by 16–18% (Osborne et al., 2014), delaying (pre)syncopes that may occur in consequence of the downward fluid shifts during the transition from hypo- to hypergravity (Goswami et al., 2012; Blaber et al., 2013). Whether the paradigm employed in this study has also substantially activated the sympathetic nervous system cannot be exclusively determined because cardiovascular data were not collected.

It is well established that sleep deprivation reduces alertness and level of attention (Lim and Dinges, 2008; Maire et al., 2018). We noted that the sleep duration and perceived sleep quality of the night before the parabolic flight were reasonable with one wake-up on average. The effect of sleep debt that we expected prior to the experiment remained moderately. However, the effect of sleep during the nights prior to the parabolic flight experiment remains to be confirmed by quantitative measurements such as actigraphy, overnight echocardiography or electroencephalography. To the best of our knowledge, no studies on sleep debt related to attention and cognition in parabolic flights have been published previously.

Our participants presented a low sensitivity level of intrinsic motion sickness susceptibility, a low level of trait anxiety, and volunteered for a parabolic flight, making a natural “selection” of participants (Collado et al., 2014, 2018; Montag et al., 2016). A lower motion sickness susceptibility of parabolic flight participants compared to control subjects was already reported in a previous study by Golding et al. (2017). However, all participants of the present study agreed to be preventively treated for motion sickness at a dose of 0.7 mL, i.e., 0.175 mg, of scopolamine that was administered subcutaneously. Only a single participant felt moderately sick during the flight after inflight data collection had been completed. To evaluate performance degradations in response to scopolamine side effects such as reduced arousal and fatigue, we assessed participant’s vigilance and sustained attention. Thirty minutes after scopolamine injection, we observed significant slower RT of the PVT, whereas the RT of the CPT only tended to increase. Previous studies have investigated the effects of scopolamine under laboratory conditions, excluding parabolic flight. Rusted and Warburton (1988) reported cognitive impairments at a dose of 0.6 mg scopolamine on problem-solving, visuospatial abilities, and spatial memory. This has also been confirmed by studies of Ebert et al. (1998) and Fredrickson et al. (2008) where different increments of scopolamine doses from placebo up to 0.8 mg impinged psychomotor function and reaction time, visual learning, executive function, and working memory. Decreases in cognitive performance peaked after one to two hours after drug injection but were still observed up to 8 h later. Flicker et al. (1990) also confirmed that high doses (0.44 and 0.63 mg) affected the performance of verbal and visuospatial recall, visual recognition memory, visuospatial praxis, visual-perceptual function, and psychomotor speed. Lower doses of 0.22 mg induced only peripheral signs, but did not impair cognitive functions (Flicker et al., 1990). Similarly, Bestaven and colleagues reported no effect on reaction time in a scoring task up to 30 min after the injection of scopolamine at a dose of 0.2 mg (Bestaven et al., 2016). However, these authors reported an effect on posture and on vestibulomotor control of the lower limbs (Bestaven et al., 2016), which could be related to the effect of scopolamine on the brainstem reported in animals (Gall et al., 2007). We found slower RT in CPT and PVT, whereas only the latter reached the level of statistical significance. Increases in RT of CPT were related to a higher *Hit Rate*, suggesting a speed-accuracy tradeoff after scopolamine administration. The differences between the PVT and CPT performance in response to the scopolamine could also be related to the high sensitivity of the PVT to wakefulness (Lim and Dinges, 2008). However, we also critically acknowledge that this discrepancy may also be the result of data acquisition under different experimental conditions using different technologies that were chosen due to time and hardware constraints. The CPT was performed in a controlled free-floating supine position with an immersive setup that minimized external visual distractors on a 15-in screen, whereas the PVT was performed in a seated position on a wrist-worn diving computer equipped with a 2-in display. Because of these considerable technical and methodological differences, the comparison of these data warrants some caution.

## Conclusion

Taken together, our data show that the experimental setting of the parabolic flight results in a significant performance decline in a Go/No-Go Continuous Performance Task, which is further aggravated during weightlessness. We attribute these findings to increased stress and anxiety state prior to the flight, and altered vestibular input related to cognitive functions including self-perception and spatial orientation during the different gravity conditions in-flight. Anti-motion sickness medication with a low dose of subcutaneous scopolamine affected slightly RT of the PVT, but not CPT. Our results indicate that it is important to control for contributing factors such as participants’ emotional state, sleep quality, and medication when designing behavioral research for parabolic flight experiments. Additionally, the control condition of 1 g should be administered as time coherent as possible with hypo- and hypergravity conditions as the impact of the contributing factors can vary throughout the flight and experiment. Future studies in larger samples are needed to verify whether the observed effects are limited to first-time flyers and to investigate potential sex-specific differences.

## Data Availability Statement

The acceleration and behavioral data that support the findings of this study are freely available on the Open Science Framework (OSF) (https://osf.io/kxn9v/).

## Ethics Statement

The studies involving human participants were reviewed and approved by Comité de Protection des Personnes Nord Ouest III, Caen, France. The participants provided their written informed consent to participate in this study. Written informed consent was obtained from the individual(s) for the publication of any potentially identifiable images or data included in this article.

## Author Contributions

AF-W and SB wrote the manuscript. M-LM, SB, and CB designed the experiment. YL was in charge of the rack and technical set up. M-LM, CB, and BP performed the data collection. AF-W performed the statistical analyses. MH coordinated, supervised, and financed saliva processing. ACS, KB, and CB revised the manuscript. All authors have discussed and interpreted the results and contributed to the final version of the manuscript.

## Conflict of Interest

The authors declare that the research was conducted in the absence of any commercial or financial relationships that could be construed as a potential conflict of interest.

## Footnotes

1 https://www.airzerog.com

2 During this phase, the aircraft flies a parabolic arc producing a freefall wherein the acceleration of the aircraft cancels the acceleration due to gravity along the aircraft vertical z-axis. With respect to an external fixed reference frame, the aircraft and all passengers and objects inside of the plane fall together with an acceleration of 9.81 m/s^2^. There is no reaction force on the passengers by the aircraft and a net level of 0 g is achieved generating the perception of microgravity or zero-gravity (Karmali and Shelhamer, 2008). Accordingly, the terms micro-, hypo-, and zero-gravity are technically incorrect because gravity is still 1 g during the entire flight maneuver. However, in the manuscript we follow the commonly used terminology in Space Life Science and use the terms interchangeably to describe a net level of 0 g during the freefall.
