# Supplementary material for: Impaired Attentional Processing During Parabolic Flight
Source: Front Physiol. 2021 May 13;12:675426. doi: 10.3389/fphys.2021.675426 (PMC8155259; doi:10.3389/fphys.2021.675426)
Supplement: Supplementary file 1 [file Data_Sheet_1.docx]

**Supplementary Materials:**

# Impaired attentional processing during parabolic flights

Friedl-Werner, A. ^1,2^, Machado, M.L. ^2^, Balestra C.^3,4^, Liegard, Y ^2^., Philoxene B. ^2^, Brauns, K. ^1^, Stahn A.C. ^1,5^, Hitier, M. ^2,6,7^, Besnard, S. ^2,8^

^1^Charité – Universitätsmedizin Berlin, a corporate member of Freie Universität Berlin, Humboldt-Universität zu Berlin, and Berlin Institute of Health, Institute of Physiology, Charitéplatz 1, CharitéCrossOver, Virchowweg 6, 10117 Berlin, Germany

^2^Université de Normandie, INSERM U 1075 COMETE, Caen, France

^3^Environmental, Occupational & Ageing “Integrative Physiology” Laboratory, Haute Ecole Bruxelles-Brabant, Brussels, Belgium

^4^DAN Europe Research Division (Roseto (It)-Brussels (B))

^5^Unit of Experimental Psychiatry, Department of Psychiatry, Perelman School of Medicine at the University of Pennsylvania, Philadelphia, PA 19104, USA

^6^Centre Hospitalier Universitaire de Caen Normandie, Department of Otolaryngology Head and Neck Surgery, Caen, France

^7^Université de Normandie, Department of Anatomy, Caen, France

^8^Aix Marseille Université, CNRS, UMR 7260, Laboratoire de Neurosciences Sensorielles et Cognitives - Equipe Physiopathologie et Thérapie des Désordres Vestibulaires, Marseille, 13000 France, phone: +33 677978649, +33 23106 53 32

**Supplementary Tables**

**Table S1**. Acceleration data of the hypogravity phases for parabolas 11 to 15 and 26 to 30 for each flight day and for each axis respectively.

|  | **Flight day I** | | | **Flight day II** | | | **Flight day III** | | |
| --- | --- | --- | --- | --- | --- | --- | --- | --- | --- |
|  | x-axis | y-axis | z-axis | x-axis | y-axis | z-axis | x-axis | y-axis | z-axis |
| **P11** | -0.013  (-0.037 – 0.012) | -0.004  (-0.014 – 0.006) | 0.006  (-0.022 – 0.037) | -0.010  (-0.035 – 0.020) | -0.004  (-0.014 – 0.006) | 0.002  (-0.029 – 0.078) | -0.010  (-0.031 – 0.010) | -0.005  (-0.016 – 0.002) | 0.003  (-0.023 – 0.090) |
| **P12** | -0.011  (-0.035 – 0.008) | -0.004  (-0.020 – 0.006 | 0.006  (-0.023 – 0.094) | -0.011  (-0.037 – 0.016) | -0.005  (-0.016 – 0.008) | 0.008  (-0.014 – 0.065) | -0.010  (-0.031 – 0.012) | -0.005  (-0.018 – 0.002) | 0.008  (-0.016 – 0.096) |
| **P13** | -0.010  (-0.031 – 0.013) | -0.003  (-0.012 – 0.004) | 0.009  (-0.006 – 0.074) | -0.010  (-0.031 – 0.016) | -0.004  (-0.014 – 0.006) | -0.005  (-0.027 – 0.078) | -0.011  (-0.035 – 0.012) | -0.005  (-0.016 – 0.002) | 0.010  (-0.012 – 0.084) |
| **P14** | -0.010  (-0.035 – 0.014) | -0.004  (-0.016 – 0.002) | 0.005  (-0.012 – 0.090) | -0.009  (-0.037 – 0.016) | -0.004  (-0.010 – 0.002) | 0.006  (-0.004 – 0.080) | -0.010  (-0.031 – 0.008) | -0.005  (-0.022 – 0.008) | 0.004  (-0.014 – 0.084) |
| **P15** | -0.009  (-0.033 – 0.016) | -0.004  (-0.020 – 0.010) | 0.004  (-0.012 – 0.096) | -0.008  (-0.027 – 0.016) | -0.005  (-0.010 – 0.000) | 0.000  (-0.027 – 0.078) | -0.009  (-0.029 – 0.020) | -0.004  (-0.021 – 0.006) | 0.009  (-0.010 – 0.066) |
| **P26** | -0.011  (-0.039 – 0.012) | -0.003  (-0.018 – 0.012) | -0.002  (-0.027 – 0.059) | -0.009  (-0.027 – 0.016) | -0.003  (-0.012 – 0.006) | 0.009  (-0.010 – 0.074) | -0.008  (-0.037 – 0.022) | -0.003  (-0.010 – 0.008) | 0.002  (-0.023 – 0.082) |
| **P27** | -0.011  (-0.035 – 0.012) | -0.005  (-0.018 – 0.006) | 0.001  (-0.012 – 0.059) | -0.009  (-0.033 – 0.012) | -0.003  (-0.014 – 0.006) | 0.006  (-0.016 – 0.082) | -0.009  (-0.031 – 0.020) | -0.002  (-0.014 – 0.012) | 0.004  (-0.014 – 0.088) |
| **P28** | -0.006  (-0.027 – 0.022) | -0.002  (-0.006 – 0.006) | 0.007  (-0.025 – 0.074) | -0.009  (-0.033 – 0.016) | -0.003  (-0.010 – 0.004) | 0.004  (-0.021 – 0.084) | -0.009  (-0.035 – 0.018) | -0.002  (-0.010 – 0.006) | 0.005  (-0.008 – 0.059) |
| **P29** | -0.010  (-0.031 – 0.016) | 0.000  (-0.018 – 0.014) | 0.006  (-0.010 – 0.090) | -0.010  (-0.037 – 0.018) | -0.004  (-0.012 – 0.006) | 0.008  (-0.018 – 0.082) | -0.008  (-0.029 – 0.018) | -0.002  (-0.012 – 0.004) | 0.005  (-0.014 – 0.066) |
| **P30** | -0.007  (-0.027 – 0.018) | -0.004  (-0.014 – 0.008) | 0.001  (-0.033 – 0.078) | -0.010  (-0.033 – 0.018) | -0.004  (-0.010 – 0.004) | -0.006  (-0.039 – 0.080) | -0.009  (-0.029 – 0.014) | -0.003  (-0.012 – 0.004) | 0.007  (-0.012 – 0.094) |

P, number of parabola; x-axis, Gx acceleration from tail to front of the aircraft; y-axis, Gy acceleration from left to right wings; z-axis, Gz acceleration from floor to ceiling. Data is presented as mean (averaged over 22 seconds) and range (minimum – maximum) for each parabola when participants completed a continuous performance task.

**Table S2**. Contrasts comparing the different points in time (Pre-flight, 1 g before P0, 0 g, 1 g after P30, and Post-flight) on CPT performance characteristics (Reaction Time, Hit Rate, False Alarm Rate, d’).

| **Contrast** | **Variable** | **Estimate** | **SE** | **DF** | ***t*** | ***P*** | **Effect Size (95% CI)** |
| --- | --- | --- | --- | --- | --- | --- | --- |
| 1 g before P0 - Pre-flight | RT | 91.00 | 30.5 | 43.2 | 2.98 | 0.019 | 0.86 (0.18, 1.52) |
|  | Hits | -6.43 | 2.2 | 43.1 | -2.93 | 0.016 | -0.85 (-1.50, -0.17) |
|  | FA | 4.49 | 1.2 | 42.9 | 3.66 | 0.002 | 1.06 (0.33, 1.76) |
|  | d’ | -0.57 | 0.1 | 42.9 | -4.31 | <0.001 | -1.25 (-1.99, -0.47) |
| 0 g - Pre-flight | RT | 78.58 | 30.5 | 43.2 | 2.58 | 0.040 | 0.74 (0.09, 1.37) |
|  | Hits | -11.19 | 2.2 | 43.1 | -5.11 | <0.001 | -1.47 (-2.29, -0.63) |
|  | FA | 5.77 | 1.2 | 42.9 | 4.71 | <0.001 | 1.36 (0.55, 2.14) |
|  | d’ | -0.87 | 0.1 | 42.9 | -6.54 | <0.001 | -1.89 (-2.84, -0.91) |
| 1 g after P30 -  Pre-flight | RT | 4.57 | 31.2 | 43.7 | 0.15 | 0.919 | 0.04 (-0.52, 0.61) |
|  | Hits | -2.58 | 2.2 | 43.7 | -1.15 | 0.514 | -0.33 (-0.91, 0.26) |
|  | FA | 0.66 | 1.3 | 43.3 | 0.52 | 1.000 | 0.15 (-0.42, 0.72) |
|  | d’ | -0.19 | 0.1 | 43.3 | -1.43 | 0.321 | -0.41 (-0.99, 0.19) |
| Post-flight -  Pre-flight | RT | 22.75 | 30.5 | 43.2 | 0.75 | 0.919 | 0.22 (-0.36, 0.78) |
|  | Hits | -1.61 | -2.5 | 43.1 | -0.73 | 0.514 | -0.21 (-0.78, 0.37) |
|  | FA | 0.43 | 1.2 | 42.9 | 0.35 | 1.000 | 0.10 (-0.47, 0.67) |
|  | d’ | -0.13 | 0.1 | 42.9 | -0.98 | 0.332 | -0.28 (-0.85, 0.30) |
| 0 g - 1 g before P0 | RT | 12.42 | 30.5 | 43.2 | -0.41 | 0.686 | -0.12 (-0.68, 0.45) |
|  | Hits | -4.76 | 2.2 | 43.1 | -2.17 | 0.099 | -0.63 (-1.24, 0.01) |
|  | FA | 1.28 | 1.2 | 43.3 | 1.05 | 0.301 | 0.30 (-0.28, 0.87) |
|  | d’ | -0.29 | 0.1 | 42.9 | -2.24 | 0.030 | -0.65 (-1.26, -0.01) |
| 1 g after P30 -  1 g before P0 | RT | -86.43 | 31.2 | 43.7 | -2.77 | 0.025 | -0.80 (-1.44, -0.13) |
|  | Hits | 3.85 | 2.2 | 43.7 | 1.72 | 0.099 | 0.50 (-0.12, 1.09) |
|  | FA | -3.83 | 1.3 | 43.3 | -3.05 | 0.008 | -0.88 (-1.54, -0.19) |
|  | d’ | 0.37 | 0.1 | 43.3 | 2.78 | 0.017 | 0.80 (0.13, 1.44) |
| Post-flight -  1 g before P0 | RT | -68.25 | 30.5 | 43.2 | -2.24 | 0.061 | -0.65 (-1.26, -0.01) |
|  | Hits | 4.82 | 2.2 | 43.1 | 2.20 | 0.099 | 0.64 (0.00, 1.25) |
|  | FA | -4.05 | 1.2 | 42.9 | -3.31 | 0.006 | -0.96 (-1.63, -0,25) |
|  | d’ | 0.43 | 0.1 | 42.9 | 3.32 | 0.006 | 0.96 (0.25, 1.63) |
| 1 g after P30 -  0 g | RT | -74.01 | 31.2 | 43.7 | -2.37 | 0.067 | -0.68 (-1.30, -0.04) |
|  | Hits | 8.61 | 2.2 | 43.7 | 3.84 | <0.001 | 1.11 (0.37, 1.82) |
|  | FA | -5.11 | 1.3 | 43.3 | -4.07 | <0.001 | -1.17 (-1.90, -0.41) |
|  | d’ | 0.66 | 0.1 | 43.3 | 4.95 | <0.001 | 1.43 (0.60, 2.23) |
| Post-flight - 0 g | RT | -55.83 | 30.5 | 43.2 | -1.83 | 0.148 | -0.53 (-1.12, 0.09) |
|  | Hits | 9.58 | 2.2 | 43.1 | 4.37 | <0.001 | 1.26 (0.48, 2.02) |
|  | FA | -5.34 | 1.2 | 42.9 | -4.35 | <0.001 | -1.26 (-2.01, -0.47) |
|  | d’ | 0.73 | 0.1 | 42.9 | 5.56 | <0.001 | 1.61 (0.72, 2.46) |
| Post-flight -  1 g after P30 | RT | 18.18 | 31.2 | 43.7 | 0.58 | 1.000 | 0.17 (-0.41, 0.73) |
|  | Hits | 0.97 | 2.2 | 43.7 | 0.43 | 0.668 | 0.12 (-0.45, 0.69) |
|  | FA | -0.22 | 1.3 | 43.3 | -0.18 | 1.000 | -0.05 (-0.62, 0.52) |
|  | d’ | 0.06 | 0.1 | 43.3 | 0.47 | 0.638 | 0.14 (-0.43, 0.70) |

RT, Reaction Time of target stimuli in ms; Hits, Hit Rate (correct reactions to target stimuli) in percentage; FA, False Alarm Rate (reactions to non-targets) in percentage; d’, indicator for task sensitivity. n = 12. *df*, degrees of freedom, *t*, t-ratio; *P*, p-value; Effect size is Cohen’s *d* and the corresponding 95% confidence interval (95% CI). Data is presented as marginal means ± SE and was collected at the following points in time: Pre-flight (30 min after scopolamine injection), inflight at 1 g before the first parabola (1 g before P0), during microgravity (0 g), at 1 g after the last parabola (1 g after P30), and after landing (Post-flight).
